# Complexity and function of family involvement in advance care planning: A qualitative study of perspectives from people living with advanced cancer, family members and healthcare professionals

**DOI:** 10.1177/02692163231194202

**Published:** 2023-09-18

**Authors:** Megumi Kishino, Jonathan Koffman, Hiroaki Nagatomi, Misuzu Yuasa, Clare Ellis-Smith

**Affiliations:** 1 Cicely Saunders Institute of Palliative Care, Policy and Rehabilitation, Florence Nightingale Faculty of Nursing, Midwifery and Palliative Care, King’s College London, London, UK; 2 Wolfson Palliative Care Research Centre, Hull York Medical School, Hull, UK; 3 Department of Nursing, Kakogawa Central City Hospital, Kakogawa, Japan; 4 Hospice Division, Seirei Mikatahara General Hospital, Hamamatsu, Japan

**Keywords:** Advance care planning, family involvement, cancer, qualitative study

## Abstract

**Background:**

Family members can support advance care planning conversations. However, how family involvement in advance care planning operates to achieve goal-concordant care remains unclear.

**Aim:**

To explore how family involvement impacts the process of advance care planning for advanced cancer patients and their family members to achieve goal-concordant care in Japan.

**Design:**

Qualitative study incorporating semi-structured in-depth interviews with thematic analysis informed by Family Systems Theory.

**Setting/participants:**

Medical oncology departments at two tertiary hospitals in Japan. A purposive sample of 13 advanced cancer patients, 10 family members and 9 healthcare professionals who cared for them.

**Results:**

Twenty-five interviews were conducted, comprising 7 dyads of patients and their family members and 18 individual interviews. Four themes were identified: characteristics of patients and family members and their views on illness and advance care planning; family context and communication; interactions with healthcare professionals and societal and cultural influences; and family members’ acceptance, preparation and confidence. Family involvement was observed as being variable at an individual level and also across generations. Family members provided patients with the instrumental and emotional support that facilitated the advance care planning process. Family involvement enabled family members to better prepare for realising patients’ wishes. It increased family members’ confidence in surrogate decision-making.

**Conclusions:**

Two mechanisms of how family involvement may enable goal-concordant care were identified: family members’ support provision and their preparation for realising patients’ wishes. Healthcare professionals should assess family’s readiness to engage in advance care planning, and the time required to prepare them for the process.

**What is already known about the topic?**

- Advance care planning has potential benefits including improved patient-centred goal concordant care, improved communication between patients and healthcare professionals and reduced decisional conflict within a family.
- Cancer is a leading cause of death globally. Evidence is required concerning the process of delivering advance care planning to patients with cancer and their families.
- Whilst family members may support advance care planning conversations, little research has focused on how their involvement in advance care planning operates to achieve goal-concordant care.

**What this paper adds?**

- Two mechanisms of family involvement in advance care planning to achieve goal-concordant care are present. First, the presence of family members can facilitate advance care planning process by providing cancer patients with instrumental and emotional support. Second, family members can better prepare for achieving cancer patients’ wishes.
- How cancer patients and their family members value family involvement in advance care planning is complex and varied. It is dependent on the family members involved and the generations of family members who participate.

**Implications for practice, theory or policy?**

- The findings from this study of how family involvement potentially achieves goal-concordant care in advance care planning inform and refine an existing logic model for a family-integrated advance care planning intervention.
- Healthcare professionals should assess family members individually and a family as a whole. This is essential to enable healthcare professionals to evaluate how the family functions, particularly concerning the patient they are caring for, and to optimise their family function.
- Cancer patients and their family members want healthcare professionals to actively engage with and collaborate across disciplines to facilitate family involvement within the advance care planning process.

## Background

Advance care planning is a process that supports adults to consider and share their values, goals and preferences regarding future care with their families and healthcare professionals so that their wishes can be respected, even when they lose the capacity for decision-making.^1^ Research has shown advance care planning may be associated with benefits. These include improved concordance between patients’ preferences and received end-of-life care, improved communication between patients and healthcare professionals, and reduced decisional conflict within a family,^2–4^ particularly where uncertainty is present.^5^

Although barriers to advance care planning implementation have been identified,^2,6^ research suggests people may be more willing to have conversations about this subject if they believe it will benefit their family members.^3,7–9^ A systematic review of surrogate decision-making across four countries, principally the United States, reported in 90% of cases where patients have lost the capacity to make decisions, family members play a key role as surrogate decision-makers in the care and treatment of patients.^10^ However, patients sometimes expect their family members to be aware of their wishes despite not having discussed them^11^ with potentially negative consequences; with one-third of surrogate decision-makers incorrectly predicting patient treatment preferences.^12^ Moreover, surrogate decision-makers may experience emotional burden as a consequence of the surrogate decision-making process.^13^ Greater support for family involvement may encourage more people to engage meaningfully in the advance care planning process. This may facilitate goal-concordant care and reduce surrogate decision-making burden.^14^ However, family dynamics are often complex and honest communication can be challenging with the potential for conflict.^14,15^ How to best involve family members remains unclear and may vary across different societies.

In Japan, palliative care has continued to develop since the Cancer Control Act affirmed it in 2007 and then in 2012 placed additional emphasis on the importance of early integration of palliative care in cancer treatment. As of 2022, the national network that comprises over 400 designated cancer hospitals is now required by the Cancer Control Plan, which is based on the law and revised in 2017, to engage in advance care planning. The Ministry of Health, Labour and Welfare has developed a national guideline on end-of-life decision-making. Alongside this, an education programme for healthcare professionals was also launched. The education programme aimed to support healthcare professionals’ understanding of the guideline, including delivering advance care planning. Healthcare professionals of all disciplines have begun to adopt advance care planning in clinical practice, but it is far from widely implemented according to the project manager of the government-commissioned education programme (Y. Kizawa, personal communication, April 18, 2023). In Japan, family involvement in conversations that include breaking bad news to patients is important.^16,17^ Moreover, family-centred decision-making in end-of-life care is considered to be the norm for many people.^18,19^ The guideline emphasises the role of family members in surrogate decision-making. However, no laws exist that support surrogate decision-making.

This study aimed, therefore, to explore experiences and perceptions of family involvement in advance care planning for advanced cancer patients, their family members and healthcare professionals to understand how family involvement operates to achieve goal-concordant care.

## Method

### Design

A cross-sectional qualitative component as part of a wider multi-method study aimed at developing and examining the feasibility of a family-integrated advance care planning intervention.

The philosophical approach underpinning this study is represented by pragmatism.^20,21^ Specifically, a pragmatic research approach is based on the proposition that the philosophical and methodological approaches should serve the question being investigated. We consider using both quantitative and qualitative methods as being complementary and necessary to address the aim of the wider study. In this component of the study, a qualitative approach was used to provide an in-depth understanding of research participants’ realities, perceptions and perspectives regarding family involvement in advance care planning.^22^ This study is reported following the Consolidated Reporting of Qualitative Research checklist.^23^

### Setting

The study was conducted in Japan where cancer accounted for 27.3% of all deaths in 2019^24^ and where the proportion of people aged over 65 years of age is currently 28.4% and is continuing to grow in size.^25^ Advance care planning is still a relatively new concept in Japan. The study was conducted in the medical oncology department of two tertiary hospitals in the central part of Japan; Nippon Medical University Musashikosugi Hospital and Central Hospital of the National Centre for Global Health and Medicine who cared for 1093 and 1542 cancer patients respectively in 2020 according to Hospital-based Cancer Registry.^26^

### Governance

Ethical approval was obtained from Research Ethics Committee at King’s College London (HR/DP-20/21-21815) and the two study sites.

### Sampling

Advanced cancer patients, their family members and healthcare professionals were included in the interviews to understand the perspectives from multiple realities.^27^ Study inclusion criteria comprised incurable advanced cancer patients and their family members who were nominated by cancer patients and aged over 20 years old. Healthcare professionals from different disciplines who had experience in advance care planning with cancer patients and their family members were also included. Table 1 presents details of inclusion and exclusion criteria. Purposive sampling was performed to reflect characteristics relevant to the study including cancer site, age, and gender. For this study, family is defined as ‘who they say they are’ irrespective of any legal relationship.^28^

**Table 1.** Inclusion and exclusion criteria for this study.

	Cancer patients	Family members	Healthcare professionals
Inclusion	• Diagnosed with incurable advanced cancer • Twenty years old or above • No cognitive impairment as judged by the attending physician • Able to provide written/recorded informed consent	• A family member (regardless of legal relationship) of a patient with incurable advanced cancer • Twenty years old or above • Able to provide written informed consent	• Physicians, nurses, medical social workers, and other occupations • Had advance care planning discussion that involves family members in their usual practice • Able to provide written informed consent
Exclusion	• Assessed as not eligible for the study by their attending physician (e.g. too ill, psychological issues)		• None

### Recruitment

The researcher (MK) explained the research to study site leads. They then introduced the study on our behalf to potential participants and asked them about their willingness to be interviewed. With their permission, the details of those who expressed interest were shared with the researcher who contacted them and provided a detailed explanation alongside a participant information sheet. All participants provided written informed consent for study participation. Recruitment was terminated when data saturation was confirmed.

### Data collection

The interview topic guides were informed by a review of the literature^29–31^ and discussions with members of the research team (JK and CE-S). Face-to-face interviews were conducted in a private room in the hospital. Cancer patients and their family members were given the choice to participate individually or together as a dyad. The dyad comprised the patient and one or more of their family member(s). Healthcare professionals participated individually. Interviews were conducted by a Japanese female researcher (MK) with a background in cancer nursing and qualitative research training and experience. The researcher had no prior relationship with the participants. Interviews were audio-recorded, transcribed verbatim in Japanese and anonymised before analysis. Field notes were taken during interviews to record non-verbal expressions from participants.^32^

### Patient and public involvement

We worked with patient and public involvement members to inform and strengthen our recruitment, data collection, analysis and interpretation of the results. The group comprised two members who have/had lived experience of cancer, either as a patient or family member and who expressed interest in supporting this study.

### Analysis

Interviews were analysed using thematic analysis which represents a systematic framework for coding qualitative data.^33–35^ Codes and themes were identified inductively from the data, followed by deductive analysis informed by Family Systems Theory.^28,36^ This theory attempts to understand the family by focussing on interactions within the family and between the family and other systems (Table 2). Family Systems Theory provided a theoretical framework to depict interactions between advanced cancer patients and their family members and interactions between the family and larger systems (suprasystem).

**Table 2.** Key concepts of Family Systems Theory guiding this study.^28^

**A family system is part of a larger system (suprasystem) and consists of many systems (subsystems).** - Each higher level system encompasses lower level systems. A family system is part of a larger suprasystem (e.g. neighbours, religious communities and healthcare system) and is composed of many subsystems (e.g. parent-child, marital and sibling subsystems). Each system can be defined and understood by its boundaries between itself and other systems.
**The family as a whole is greater than the sum of its parts.** - How these parts relate, communicate and behave is important to the overall understanding of the family as a whole. This also indicates that individuals are best understood within a larger system, for example, the family.
**A change in a family member affects other family members.** - As a family is seen as an interactive unit, no individuals exist in isolation within a family. Therefore, any significant event or change, including a diagnosis of a life-threatening condition in one family member affects all family members to varying degrees.
**The family can create a balance between change and stability.** - There used to be two polarised views of families. The first belief was that families tend towards maintaining equilibrium, then another belief families are always changing came instead. However, von Bertalanffy suggested that change and stability can coexist in living systems. This may be achieved by a reorganisation of the family in ways that are different from the ways how they used to be organised before diagnosis.
**Each family member’s behaviours are best understood from a view of circular causality than linear causality.** - One method of understanding a family is to observe patterns within a family. There is a difference between linear and circular patterns. Linear causality is a relationship in which one event (A) causes another (B), whereas circular causality explains B also affects A which subsequently results in A affecting B again.

Data were managed with NVivo software. Analysis from the first four interviews was discussed with the second researcher (HN), a Japanese male nurse who specialised in family nursing which guided the analysis of the following interviews. All transcripts were analysed by the researcher (MK). The second researcher (HN) reviewed all of the analyses. When disagreement occurred a third researcher (MY), a Japanese female family physician with expertise in advance care planning was involved. Field notes and a reflexive diary aided data interpretation.^32^ ‘Member checking’ with study participants to validate interview transcripts or analysis did not take place. However, themes, codes and key quotations were shared with the patient and public involvement members who represented cancer patients and their family members to authenticate the analysis and find new themes.^37^ Themes, codes and key quotations were translated from Japanese into English by the researcher (MK) and then discussed with members of the study team in the UK (JK and CE-S). The final themes and illustrative quotations were translated into Japanese by the third researcher (MY) and then confirmed by the researcher (MK). Table 3 describes the measures we took for analytical rigour.^38–40^

**Table 3.** Measures taken to ensure analytical rigour.^38–40^

Quality criteria	How it was achieved
*Rich rigour* – Analysis uses appropriate sample, context and data driven by theory	We interviewed 13 people with advanced cancer, 10 family members and 9 healthcare professionals having experience in advance care planning conversations with people with advanced cancer. Semi-structured interviews were performed using topic guides which were developed to facilitate participants to share their views on family involvement in advance care planning. We drew on Family Systems Theory to deductively analyse the data.
*Credibility and authenticity* – Thick descriptions and detailed findings have been provided to support inferences	We included healthcare professionals and we drew on multiple perspectives to provide a more comprehensive picture of the situation. We selected quotations from a range of participants across all three participant groups.
*Criticality* – A detailed account of how researchers critically appraised their findings	The researcher (MK and HN) responsible for data analysis openly and frankly discussed potential assumptions concerning advance care planning and family throughout the analysis and developed the themes. These themes and illustrative quotations were shared with and obtained feedback from patient and public involvement members which comprises people with cancer and their family members to reduce healthcare professionals’ bias.
Attention to contradictory or non-confirmatory data	During analysis, the researcher paid attention to data that contradicted emerging themes. Field notes taken during the interviews and a reflective diary which the researcher kept during data collection and analysis supported the process by providing more information about the context.
*Fidelity or meaningful coherence* – Analysis achieves its intended goals by using appropriate methods	A systematic review previously conducted in our project and the developed logic model^13^ supported the researcher to focus on ‘family involvement’ in advance care planning. This helped informed what to explore when developing topic guides and conducting interviews, and what are the similarities and differences compared to the previous studies when analysed.

## Results

Interviews were conducted between October 2021 and January 2022. Seventeen cancer patients, 14 family members and 9 healthcare professionals were approached. Seven people declined to be interviewed. The reason for this included being too unwell (*n* = 1), a cancer patient not wishing for their family member’s involvement in the interview (*n* = 1), being too busy (*n* = 1), and feeling uncomfortable talking about the topic (*n* = 1). One family, a cancer patient and two family members did not provide the reason. One cancer patient consented but subsequently did not attend the interview due to health reasons. A total of 13 cancer patients, 10 family members and 9 healthcare professionals were successfully interviewed. This included seven dyads comprising the patient and their family member. The mean interview time was 44 min (range 12–69 min). Table 4 presents the characteristics of the study participants.

**Table 4.** Characteristics of study participants.

Characteristics	*n*
Cancer patients	13
Age	
Median (range)	66 (52–81)
Gender	
Male	5
Female	8
Primary cancer site	
Breast	7
Colorectal	2
Gynaecological	1
Renal	1
Lung	1
Pancreas	1
Family members	10
Age	
Median (range)	59 (46–76)
Gender	
Male	7
Female	3
Relationship to cancer patients	
Spouse	6
Adult child	2
Sibling	1
Friend	1
Living with cancer patients	
Yes	9
No	1
Healthcare professionals	9
Profession	
Physician	4
Nurse	3
Psychologist	1
Social worker	1
Years of experience	
Median (range)	23 (5–33)
Gender	
Male	3
Female	6

**Figure 1.**
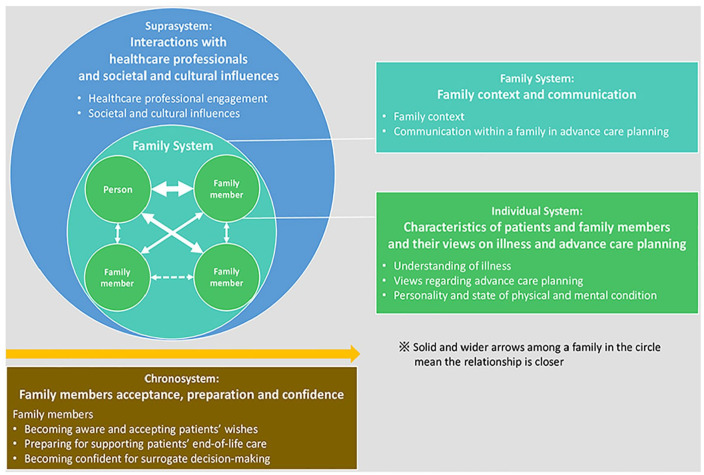
A conceptual model of functions and influence factors of family involvement in advance care planning.

We identified that the data aligned well with the levels of systems within Family Systems Theory. This enabled us to organise our themes. Specifically, four themes were identified (Figure 1); ‘Characteristics of patients and family members and their views on illness and advance care planning’, ‘Family context and communication’, ‘Interactions with healthcare professionals and societal and cultural influences’ which correspond to each system described in Family Systems Theory. The fourth theme ‘Family members’ acceptance, preparation and confidence’ explained the temporal dimension which we added to the systems in Family Systems Theory as the chronosystem.

### Characteristics of patients and family members and their views on illness and advance care planning

This theme describes individual factors that, from the perspectives of participants, appeared to influence the meaning of family involvement in advance care planning to them.

Cancer patients or family members’ understanding of their illnesses influenced their respective willingness to be involved in the advance care planning process. When family members did not understand the patient’s illness, and cancer patients were aware of this, they were reluctant to involve family members. Views regarding advance care planning shaped the extent to which cancer patients wanted to involve their family members and similarly, family members’ wishes to be included too. Views varied for some who saw less value in advance care planning, illustrated by a participant who stated, ‘*Things will take their own course* (Family 3)’ to others who stressed the importance of explicitly sharing their wishes before they lost the ability to speak for themselves. Another participant held a more ambivalent view: ‘*I can only discuss (future care) based on the current situation. If something unexpected happens all I can do is leave the decision to my family* (Patient 7)’.

The value which family involvement in advance care planning also varied. Most participants mentioned the practical reasons why it might be of use to them. For example, a cancer patient explained her decision which she believed had ramifications for her family members that included financial issues and where they lived.

. . ., when I became unable to move and had to rent a nursing bed or other personal items, I was told that it was very expensive. But if I lived in the city where I lived before it (the city) would back up 90% of the cost. So, I’ve been talking with my husband about moving to the city again. (Patient 12)

For some cancer patients, advance care planning’s value was seen as reducing the burden of surrogate decision-making for family members, irrespective of any potential benefits for themselves. A cancer patient who decided on medical treatment for his father who lacked mental capacity stated,

I hope we can discuss it beforehand. I think that families would be distressed. They are put in that kind of situation. At that time, it would be a great help if they knew that it was the person’s will, wouldn’t it? Well, even using my father’s example earlier, I think it was very difficult for me to make that decision. (Patient 8)

Another aspect related to participants’ views on family involvement in advance care planning appeared to be guided by either their own or others’ personal characteristics, for example, their personality. A husband of a cancer patient who often came to the hospital stated he should be with his wife when talking about her future care, ‘*My wife is a great worrier. Even though the doctor didn’t say anything that bad she takes it very badly. . . . I come with her, and we listen to the doctor together. And then I support her at home by trying to make her understand what was said* (Family 4)’.

Participants also emphasised the importance of focussing on current treatment and leading life to the full despite the presence of their illness. A family member shared, ‘*the advance care planning conversation is not a priority. First of all, we think about the disease, how to treat it, and things like that, because that is by far the highest priority* (Family 3)’. For many, the difficulty in experiencing cancer treatment whilst at the same time attempting to cognitively prepare for worsening illness appeared to account for why advance care planning for cancer patients was challenging. Healthcare professional participants also shared this sentiment. Moreover, some participants described that their physical and mental state, often affected by their cancer treatment, influenced their willingness and preparedness to consider future care.

### Family context and communication

This theme refers to the interactions within a family and explains how family context contributed to the complexity of family involvement in the advance care planning process. Various family relationships were evident among study participants. Participants spoke about their relationship with one another, the frequency and depth of daily conversations, sharing medical situations and how long they spent time together, all of which appeared to influence conversations regarding future care. Several described an event in the past that changed their relationship in the context of living with cancer. For example, a son explained that he had commenced accompanying his mother who had cancer to the hospital after his father died 2 years before. Whilst the legal relationship in decision-making appeared to be important to most participants the meaning of what ‘family’ implied to some was not necessarily tied to a legal or blood relationship. For example, a close friend who lived with a cancer patient and attended hospital appointments with him explained her role, ‘*I’ve been trying to share the medical situation in detail with his sister. I’m not his wife or sister, no legal or blood relationship* (Family 1)’ Participants also described family members’ various roles and situations regarding advance care planning concerning surrogate decision-making.

Sometimes a surrogate decision-maker and the people who actually know the person better are different. (Healthcare professional 4)

There were instances where family members were not present during hospital appointments that, nevertheless, still influenced decision-making. A wife of a cancer patient explained changes in the way her husband’s felt about his cancer after his 92-year-old mother passed away, ‘*While she (mother) was alive, he was very concerned if he were to die first. And now he got to see her die, and he got to see her off, so in a way, he feels, you know, free from concerns*. . . (Family 10)’. Despite their influence on decision-making, cancer patients and healthcare professionals often hesitated to involve younger or older family members, for example, elder parents or young children, in advance care planning discussions. This resulted in these family members being excluded even though healthcare professionals’ direct engagement with family members was valued by patient and family member participants in this study, as described in the next theme.

Family communication did not function well when there were conflicts or differences in opinion present within the family. This had repercussions for the advance care planning process. In some instances, conflicts resulted in family members’ opinions outweighing the patient’s opinion which hindered open and shared communication. In other cases, whilst the cancer patient was ready to talk about future care their family members were not, ‘*It’s not always a pattern of the person feeling difficult and the family being able to hold on* (Healthcare professional 8)’.

Family worked well when family members provided cancer patients with support which enabled better communication with healthcare professionals, for example, by asking questions and advocating on behalf of cancer patients. Moreover, family members supported cancer patients emotionally, for example, reassuring them and maintaining their hope.

My husband often says that medicine is ever evolving and there is always the possibility of new treatments being found. I am rather pragmatic and think about what to do in the future. Well, I think it’s good to have both. I don’t know what to do if they are both weirdly positive and if they are both very negative and talk about how I am going to die, it will interfere with our daily life. It’s balanced to have both in a way. (Patient 12)

Some support could only be offered by family members. This highlighted the benefit of family involvement which is distinctive from healthcare professionals’ support and could be provided within or outside the hospital setting. For example, when family members were intimate with cancer patients they were able to share memories and experiences. The process of sharing helped facilitate the advance care planning process by recognising and identifying values important to the patient.

The family members would help draw out the person’s thoughts and feelings that cannot be expressed. In such exchanges, there are times when the person says to their family, ‘You know me so well’. I think that because the family has lived together, they really understand these values. (Healthcare professional 1)

### Interactions with healthcare professionals and societal and cultural influences

This theme refers to family interaction with the larger systems including healthcare professionals, and cultural and societal contexts. Cancer patients and their family members regarded healthcare professionals’ direct engagement with a family in conversations positively. Conversations that involved family members were seen as an opportunity for family members to obtain information from healthcare professionals directly and establish a relationship. Healthcare professionals considered direct engagement with family members as being important. Additionally, cancer patients and family members valued healthcare professionals’ presence during advance care planning conversations since they represented an objective third party for the family, were useful in initiating conversations, and managed challenging situations that arose during communication within families. A sister of a cancer patient stated, ‘*If we limit discussions to within a family, we may get emotional. Someone who is an outsider offers reassurance* (Family 9)’.

Although cancer patients and family members desired healthcare professionals’ engagement, they were, nevertheless, unsure which professional would be best to consult with since their concerns related to advance care planning were broad; ‘*I think there are lots of things that probably bother us, but you can’t necessarily talk to someone who doesn’t understand the treatment* (Family 8)’. Healthcare professionals dealt with this through multi-disciplinary collaboration which was valued by all participant groups. For example, nurses explored the feelings and concerns of cancer patients and their family members after a physician initiated a conversation.

Participants described external societal and cultural influences on family. This included the extent to which living together and making decisions together as a family was important. However, how the family was valued appeared to differ across generations which had implications for how different members of a family engaged in the advance care planning process.

After all, the environment in which a person has been brought up has a tremendous influence on the formation of a person’s sense of values or something like that. So, when I was born, I, my great-grandfather, great-grandmother, grandfather and grandmother, my parents, were all alive. So, from the time I was about three until I graduated from primary school, my great-grandparents, grandparents all died, so I saw a lot of funerals as a child. Because we lived in the old days, in those days, we had funerals at home. It wasn’t a funeral hall like now. So, I was more familiar with it, or rather, I had that experience, so when someone dies, it’s like this is how it’s done. (Patient 7)

### Family members’ acceptance, preparation and confidence

This theme refers to changes among family members in their preparation to achieve cancer patients’ wishes. These changes occurred over time as a consequence of a series of conversations in which they increasingly shared their views among a family.

Involving family members in conversations enabled family members to become aware of the cancer patient’s wishes. It also helped family members gain a greater appreciation of the cancer patient’s views and values. In some instances, family members’ views were contradictory to patients’ wishes, however, the process of their involvement led to a joint agreement. This process of achieving joint acceptance was frequently accompanied by negotiation between the parties and often required healthcare professionals to sensitively manage family members’ emotions and concerns and provide appropriate information regarding care options and available resources.

It is impossible for the patient’s wishes to be respected unless the family is satisfied with the patient’s wishes. The family may also have their own ideas about what kind of end they want the patient to have, and unless these are balanced, I don’t think the patient will be able to have the end of life they want. So, I think it’s important, that through conversations, family members get to know the person’s wishes and the person also understands their family’s thoughts. They then reach an agreement on how the person’s final days will be and make decisions accordingly. (Healthcare professional 3)

Talking about future care prepared family members emotionally and to practically achieve the cancer patient’s wishes. The importance of early engagement appeared to be all the more important when complicated family situations required a greater time and effort to resolve before discussing values and wishes.

If a person wants to stay at home, it is difficult for them to do so without involving their family. In that sense, if you don’t get the family involved early enough and make them aware of the illness and look at it as linear rather than a dot, then, for example, suddenly, the family have more work to do and they can’t make it. (Healthcare professional 5)

Family members’ confidence in surrogate decision-making was strengthened by sharing conversations, retaining what cancer patients said throughout the process and gaining a deeper appreciation of their preferences and thoughts; ‘*When I am listening to her talking to the doctor, I don’t think I would think “Oh, I see, that’s what she thinks. I would do so for her if that happens.” But when I suddenly find myself in that (surrogate-decision making) situation, I would remember this conversation and I think it helps me convince myself that I should go in this direction* (Family 2)’. Being involved in the advance care planning process enabled family members to learn more about the cancer patient. Family members recognised that this would help them make decisions on behalf of the cancer patients should they become surrogate decision-makers which in the future would make them more sensitive to what cancer patients said during the process.

## Discussion

In this study conducted in Japan, we identified how family involvement may enable goal-concordant care in the advance care planning. This has informed the refinement of our existing logic model of a family-integrated advance care planning intervention, based on our recent systematic review.^14^ The first mechanism is represented by support from family members to facilitate cancer patients’ advance care planning. We also detail support as including assisting in communicating with healthcare professionals and providing reassurance. Notably, family members play an important role by providing support that cannot be provided by healthcare professionals alone. The second mechanism identified in this study is characterised by family members’ preparation to achieve cancer patients’ wishes. This finding is consistent with previous research regarding family meetings which led to family members’ preparation, emotional adjustment and treatment decisions that aligned with the patient’s wishes.^41^ Healthcare professionals should therefore consider involving family members taking into account the time involved in families to contribute effectively to the advance care planning process. To enable these mechanisms to operate, or to enhance family functioning, assessing a family as a whole by focussing on interactions among them is essential.

A nationwide Japanese survey of bereaved family members reported that 76% of participants stated they commenced preparation of care for the death of their dependents including ‘increasing the time spent with the patient (e.g., being together at home or in a hospital room, and travelling)’.^42^ Some family members are willing to actively prepare for the inevitable death of their dependents. Advance care planning conversations among a family and sharing patients’ wishes may help these families achieve care that aligns more closely with cancer patients’ wishes.

Whilst previous studies have explored the importance of family in advance care planning^43^ this study goes further to provide a unique understanding of the nuances and tensions that might be present and how these can be overcome through sensitive assessment and support from healthcare professionals. Specifically, we identify that family frequently comprises multiple members who may have different levels of involvement or impact in decision-making, and who may or may not be known to healthcare professionals involved in the advance care planning process. We also observed that there may be some family members who know the cancer patient best but are not always identified as being the named surrogate decision-maker involved in the advance care planning discussion. Moreover, there may be family members who directly impact on advance care planning process but are not involved, for example, dependents such as children or older relatives. Involving dependents can be challenging, particularly when there are tensions between children’s desire for agency and parental desire to shield children from the illness.^44^ However, those dependents may want to be involved in conversations and end-of-life care.^44^ Therefore, to realise cancer patients’ wishes by reducing possible conflict among a family, healthcare professionals should broach this topic sensitively and consider with the patients which family members should be present during conversations.

This study identified that the role of family involvement can be multifarious. Most participants gave practical reasons for family involvement in advance care planning. However, we also observed that the role of family involvement was understood differently across generations. This may be explained by the context in which this study was conducted. More specifically in 1947, Japan repealed the patriarchal system where families are typically headed up by a male position of authority and also that there are now many smaller households in Japan where individuals increasingly live separately from their families.^45^ Furthermore whilst only 18% of cancer patients in Japan were aware of their diagnosis in 1992 this has increased to 94% in 2020.^46,47^ Participants affirmed family involvement itself in advance care planning; however, these culturally shaped factors may affect people’s perception of ‘family’, for example, to what extent they consider the other family members’ opinions when making decisions and to what extent family members insist their views over the patient’s wishes. Moreover, cultural characteristics supported by previous studies, families’ preferences tend to be valued over patients’ own,^18^ were not evident in this study. As previous research indicated,^48^ avoiding assumptions and assessing what family involvement means and whether family involvement works in each family is crucial.

## Further development of the logic model of a family-integrated advance care planning intervention

**Figure 2.**
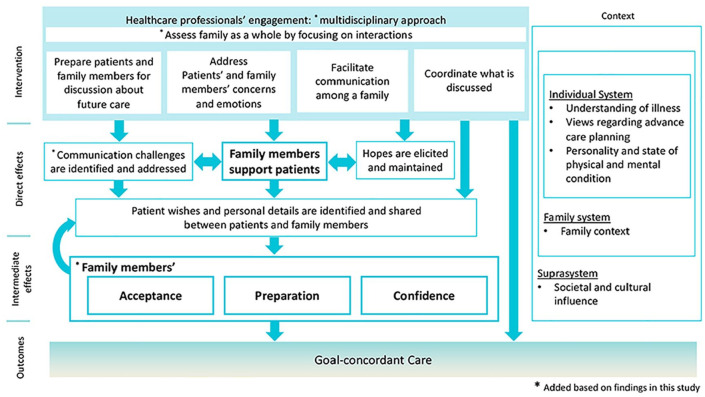
A logic model for family-integrated advance care planning intervention to achieve goal-concordant care.

Based on the findings from this study, Figure 2 progresses our previously developed logic model,^14^ informed by Rowher et al.^49^ The logic model depicts the direct and intermediate effects of family-integrated advance care planning intervention and the intended outcome, as well as contextual factors. Specifically, this study identifies three areas for refinement. First, we have incorporated the intervention components, specifically, the requirement for a multi-disciplinary approach and to assess a family as a whole. Second, we added a new element ‘communication challenges are identified and addressed’. Third, we have divided the ‘direct’ and ‘intermediate’ effects associated with goal-concordant care. Intermediate effects represent ‘family members’ acceptance, preparation and confidence’.

Despite various arguments including scepticism of advance care planning value,^50–52^ there seems to be a common ground that ‘preparing’ patients and ‘family members’ for end-of-life decision-making is crucial regardless of geographical location. Moreover, the importance of family integration in advance care planning has been gaining more attention.^2,53^ The role of the family in this process should consequently be revisited. The findings in this study that illustrated family involvement in advance care planning and explained how it operates in the process could contribute to unravelling the complexity and provide a theoretical underpinning for a family-integrated approach.

## Strengths and limitations of the study

A strength of this study is that we interviewed cancer patients either by themselves or jointly with their family members. When they were interviewed together, the dynamics of the interview elicited additional valuable perspectives, whereas when interviews were conducted with patients alone some participants felt able to share perspectives without needing to be concerned about how the others might feel about their views. We also obtained the views of healthcare professionals and were able to compare and contrast their insights with other participants’ perspectives. This study also has limitations. First, cancer patients nominated their respective family members to be study participants. We permitted this pragmatic decision considering the complexities associated with advance care planning, the sensitivities associated with those living with cancer and the needs of their family members. By abrogating this decision to cancer patients, we may have excluded other relevant family members’ perspectives. Second, people with breast cancer made up more than half of the study participants. Whilst people with this type of cancer may theoretically have held a particular worldview, we did not identify a theme specific to just them. Third, since this is a cross-sectional study, we were unable to examine how family involvement influences the advance care planning process over time. We recognise that cancer patients’ wishes may evolve over time,^40,54^ partially due to the progress of their illness and also due to changing interactions within families.^55,56^ Future studies involving sequential interviews should explore how this temporal dimension associated with family involvement in advance care planning can feasibly be captured.

## Conclusion

Two mechanisms of how family involvement in advance care planning may enable intended outcomes, specially goal-concordant care were identified. First, family members’ support provision can facilitate advance care planning process and second, they can prepare for achieving cancer patients’ wishes. How people value family involvement in advance care planning is diverse, complex and variable depending on which family members are included and across generations. Therefore, to enable these mechanisms to operate to the best effect, healthcare professionals should assess family members individually and a family as a whole. This is essential to permit healthcare professionals to appreciate how a family functions. It takes time for healthcare professionals to assess how family members communicate with each other and work together to make decisions, and to optimise how they may do so. Therefore, healthcare professionals should consider when and how much time is required to involve family members in advance care planning.
